# Conversion of Stem Cells to Cancer Stem Cells: Undercurrent of Cancer Initiation

**DOI:** 10.3390/cancers11030345

**Published:** 2019-03-11

**Authors:** Said M. Afify, Masaharu Seno

**Affiliations:** 1 Department of Medical Bioengineering, Graduate School of Natural Science and Technology, Okayama University, Okayama 700-8530, Japan; saidafify@s.okayama-u.ac.jp; 2 Division of Biochemistry, Faculty of Science, Menoufia University, Shebin El Koum-Menoufia 32511, Egypt; 3 Laboratory of Nano-Biotechnology, Graduate School of Interdisciplinary Science and Engineering in Health Systems, Okayama University, Okayama 700-8530, Japan

**Keywords:** stem cell, cancer stem cells, induced pluripotent stem cells, cancer-inducing niche, chronic inflammation

## Abstract

Cancer stem cells (CSCs) also known as cancer-initiating cells (CIC), are responsible for the sustained and uncontrolled growth of malignant tumors and are proposed to play significant roles in metastasis and recurrence. Several hypotheses have proposed that the events in either stem and/or differentiated cells, such as genomic instability, inflammatory microenvironment, cell fusion, and lateral gene transfer, should be considered as the possible origin of CSCs. However, until now, the exact origin of CSC has been obscure. The development of induced pluripotent stem cells (iPSCs) in 2007, by Yamanaka’s group, has been met with much fervency and hailed as a breakthrough discovery by the scientific and research communities, especially in regeneration therapy. The studies on the development of CSC from iPSCs should also open a new page of cancer research, which will help in designing new therapies applicable to CSCs. Currently most reviews have focused on CSCs and CSC niches. However, the insight into the niche before the CSC niche should also be of keen interest. This review introduces the novel concept of cancer initiation introducing the conversion of iPSCs to CSCs and proposes a relationship between the inflammatory microenvironment and cancer initiation as the key concept of the cancer-inducing niche responsible for the development of CSC.

## 1. Introduction

Recent studies have revealed the heterogeneity of cell types that are present within malignant tissues. As a result, tumors are comprised of a diverse collection of cells, with distinct molecular signatures and different levels of sensitivity to treatment [1].

Over the decades, there two major models that have been used to explain cancer heterogeneity: the clonal evolution model (mutation hypothesis) and the cancer stem cell model (cellular hierarchy organized in a tumor) [2,3,4].

**Figure 1:**
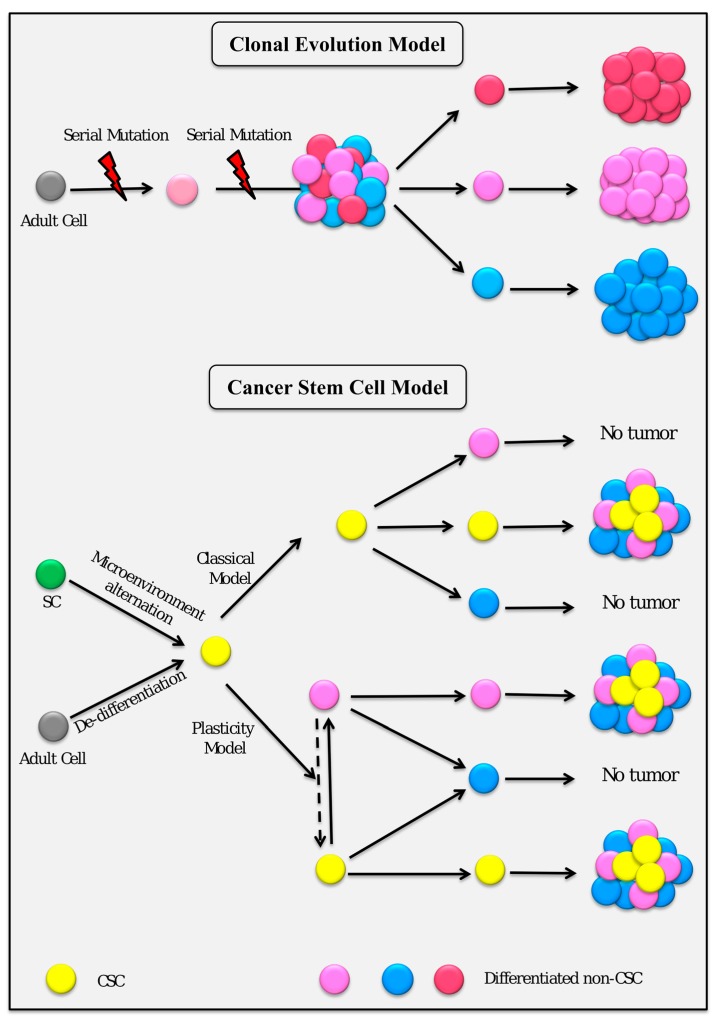
Schematic illustration for the stochastic and cancer stem cell (CSC) models of tumorigenesis.

The first attempts to understand the basis for the mutation hypothesis originated in 1914, when Boveri postulated that cancer could result from a combination of chromosomal defects [5]. After this movement in research toward understanding the biology of cancer, the DNA double helix and genetic information were discovered in the 1950s [6], which paved the way for the work of Carol O. Nordling, who suggested that a number of mutated genes could cause cancerous cells to form a tumor [7]. After this hypothesis, the number of mutational changes required to cause cancer was extensively been investigated. As a result, Ashley posited that approximately three to seven mutations might be required for the development of cancer [8]. Furthermore, Weinberg confirmed that at least three or four mutation were required for the appearance of malignant phenotypes in vitro [9]. Stochastic models have suggested that serial mutation events generated tumor cell heterogeneity and contributed to cancer progression [10]. In this model, most cancer cells should possess several mutations that give the cells malignant properties, and each mutation increases the probability of the next (Figure 1). The main concept of this theory was that cancer should result from time-dependent accumulation of DNA mutations in a single cell. Accordingly, cancers were thought to be monoclonal, i.e., they were all considered to be derived from a single mutant cell, thereby generating a homogeneous tissue composed of malignant cells [11]. Simultaneously, some scientists thought that mutations occurred in DNA, but without causing cancer. Mutational changes generally would be insufficient to cause cancer, because a minority of cancers were only triggered by about 5% mutations [12]. Others noted that some cancers were not associated with any mutations whatsoever [13,14]. On the other hand, many scientists demonstrated that carcinogenesis was a result of conversion of normal cells into CSCs.

**Figure 2:**
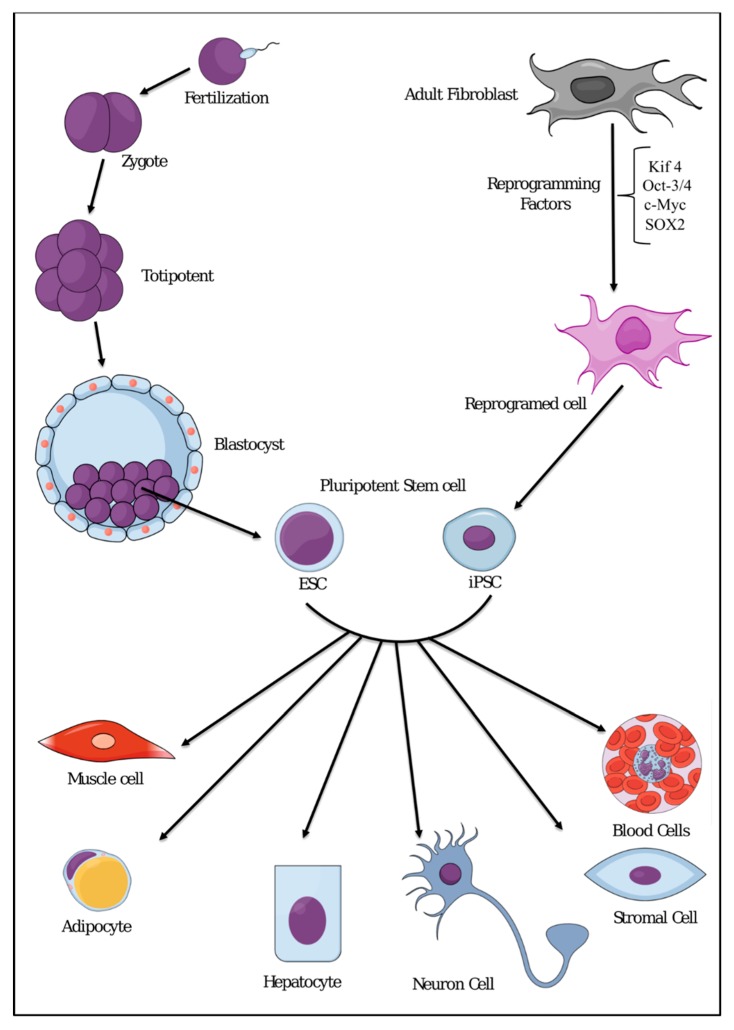
Representative scheme showing differentiation potential of pluripotent stem cells such as embryonic stem cells (ESCs) or induced pluripotent stem cells (iPSCs) into all cell types.

Normal stem cells are described as immature cells that have the double capability of self-renewal and differentiation potential [15,16,17]. Stem cells were not discovered by a specific scientist or a group, but the concept was established through the continuous effort over the past several decades by many scientists. Alexander Maksimov, a Russian histologist, who developed and introduced a theory of hematopoiesis, was the first to propose the term “stem cell” in the early 20th century [18]. Stem cells were at first believed to be present only in certain tissues, such as blood, liver, and intestinal epithelia, but nowadays they have been recognized to be present in every tissue in the body [19,20]. Immature cells were first isolated from the inner cell mass of the mouse embryo at blastocyst stage by Martin Evans and Matthew Kaufman [21] and Gail R. Martin, who named the cells “embryonic stem cell (ESC)” [22] The first isolation of human ESCs from fertilized blastocysts in vitro was done by Thomson [23]. ESCs are defined by the capability to proliferate conservation of an undifferentiated phenotype for prolonged periods [24,25], and by the pluripotency of differentiation into all lineages of the primary three germ layers, endoderm, ectoderm, and mesoderm (Figure 2) [26,27]

During the conversion of the normal stem cells into CSCs, many changes can happen, like abnormal cell division and epigenetic and genetic changes. This implies that the mutation hypothesis is not always applicable in the formation of a cancer. The conversion is considered to be the causal event in the origin of the vast majority of cancers. Most of the processes of carcinogenesis should consist of sequences of steps that initiate cancer. Somatic mutations should follow these steps as the later events occurring after carcinogenesis. Finally, somatic mutation theory seems to be inaccurate, as mutations are not always the main causal event of cancer development [28]. Since somatic mutation theory cannot explain the complexity/heterogeneity of cancer tissues, it should be dropped and replaced with another theory/hypothesis [29]. The common properties of self-renewal potential and pluripotency between CIC/CSCs and normal stem cells have led scientists to formulate a new concept for abnormal stem cell disease, instead of the mutation theory for cancer [30]. The CIC/CSC model suggests that only certain subpopulations of cancer cells have the ability to drive the progression of cancer. These are specific and more aggressive subtypes of cells, which result in tumor progression and recurrence [31]. This subset of tumor cells has the capacity to initiate and sustain tumors when transplanted into immune-compromised animal hosts, due to its self-renewal and the generation of differentiated progenies [32,33]. The differentiation of CSCs results in the cellular heterogeneity in tumors, as well as exhibiting inherent drug resistance and enhancing invasive potential, which plays a critical role in initial tumor formation and metastatic progression [34]. Therefore, CSCs should be a pivotal target for the eradication of many cancers.

According to the classical CSC hypothesis, there is a unidirectional differentiation hierarchy, where non-CSCs cannot generate CSCs (Figure 1). However, it is increasingly apparent that differentiated cells can be switched to CSCs [35]. This new hypothesis of heterogeneity opens the way for a CSC plasticity model, which supports the idea that the differentiated stage of the cells can be converted back to an undifferentiated stage or stem cell-like stage [36,37]. Collectively, CSCs could be considered as a dynamic subpopulation of cancer cells.

Enormous effort has been made to determine the origin of CSCs until now. However, little is known about the origin of CSCs in tumor tissues. The field of stem cell research has attracted more scientists than ever to study the stem cell biology and the possibility of the generation of CSCs. However, the use of ESCs in research is still controversial from an ethical point of view. Therefore, the discovery of induced pluripotent stem cells (iPSCs) opens a new page in stem cell research. iPSCs are reprogrammed from completely differentiated cells to take on the characteristics of ESCs, including the ability to give rise to all the cell types in the body (Figure 2) [38,39,40]. This reprogramming process was first established by reprogramming differentiated fibroblasts with the four stemness genes, Oct4, c-myc, Klf4, and Sox2 [41]. The scientific progress with iPSCs to date seems very promising. iPSCs are considered to be an ideal replacement for ESCs, and many efforts have been made to understand their nature. The most important contribution of iPSCs to medicine is the potential of generating stem cells for clinical applications without sacrificing embryos [42]. In this review, we try to focus on chronic inflammation, which can stimulate pluripotent stem cells or progenitor cells in each tissue to generate CSCs.

## 2. Inflammatory Microenvironment Stimulation and Cancer

The link between inflammation and cancer was first observed in the nineteenth century by German physician Rudolf Virchow [43,44]. Virchow observed leukocyte infiltration inside tumors, so he assumed that the cancer was originating at the sites of chronic inflammation. These leukocyte infiltrates were initially assumed to be merely a sign of immune responses. In the last decades, Virchow’s principles have been supported by considerable evidences that inflammation plays a critical role in tumorigenesis [45,46]. The reason why leukocytes infiltrate tumors has not been well established. What is the functional role of the leukocytes in the tumor observed by Virchow? Are they promoting tumors in place of self-protecting or wound healing? Although the link between inflammation and cancer is now largely accepted [47], the exact mechanism is not clear yet.

To understand this relationship between inflammation and cancer, we should understand first the process of inflammation. Inflammation is generally considered to be an immune reaction against a pathogen [48]. Sometimes a pathogen injures tissues, sometimes injured tissues are invaded by pathogens, and, on other occasions, immune cells that respond to pathogens even without injuries, e.g., activated immune cells producing reactive oxygen species (ROS), may injure the tissues.

The first responses of the immune system against infection, which stimulate the release of chemical factors by infected cells, establish a physical barrier against the spread of infection by clearing pathogens. Resident immune cells present in all tissues, such as macrophages, histiocytes, Kupffer cells, and mast cells, are primarily responsible for the initiation of this process. The chemical factors such as histamine, serotonin, leukotrienes, and prostaglandins produced during the process cause local vasodilation of the blood vessels and attract phagocytes, especially neutrophils [49,50]. Neutrophils then trigger other parts of the immune system by releasing factors that attract additional leukocytes and lymphocytes responsible for repairing.

**Figure 3:**
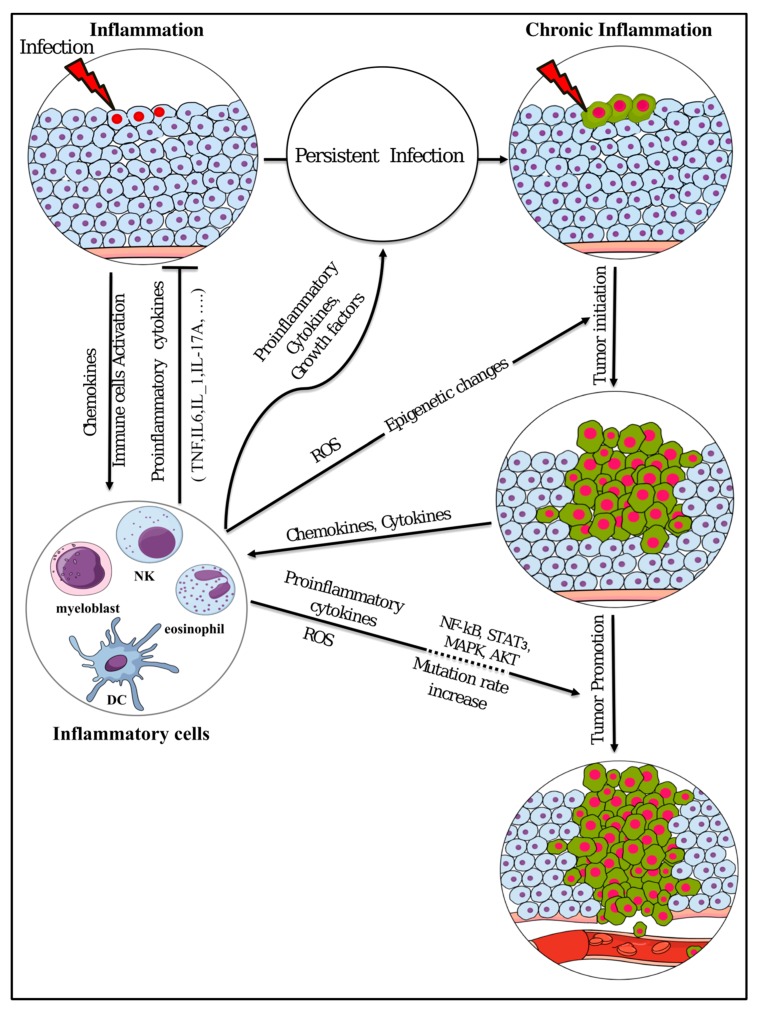
Summary of inflammation involvement in tumor initiation and promotion. ROS: reactive oxygen species; DC: dendritic cell; NK: natural killer.

Acute inflammation is usually self-limiting; however, chronic inflammation develops, resulting in organ dysfunction, when acute inflammation cannot be controlled or resolved. Chronic inflammation results from continuous infection or delayed wound healing that keeps inflammatory cells activated. These activated cells keep secreting pro-inflammatory mediators, such as cytokines and chemokines [51], which in turn initiate cancer (Figure 3) [52,53].

Chronic inflammation plays critical roles at different phases of tumor development. The initial mechanism of tumor development could be triggered by the inflammation, which may enhance the production of cytokines, chemokines, growth factors, proangiogenic factors, and extracellular matrix-modifying enzymes, which should induce signal transductions essential for cell survival and proliferation, facilitating chromosomal instability [54]. It is assumed that the production of proinflammatory cytokines, which activate transcription factors, such as NF-κB and STAT3 in pre-malignant cells, is the main tumor initiating mechanism [55,56]. Playing key roles in chronic inflammation and tumor initiation, cytokines such as TNF, IL-1, and IL-6 and activated transcription factors like NF-κB and STAT3 control the main pro-tumorigenic signaling [57,58], which induces cellular transformation and malignancy [59].

Tumor promotion is also considered to be the result of chronic inflammation through unusual epigenetic modification. It is well known that epigenetic processes can control gene expression without changes in DNA sequence [60]. These epigenetic changes occur in response to the microenvironment [61], which may change in chronic inflammation and lead to unusual epigenetic modification, causing irregular activation or silencing of genes. Collectively, the unusual epigenetic modifications are considered to play critical roles in all types of cancers [62]. Taking these into consideration, chronic inflammation should critically contribute to tumor initiation and promotion by changing epigenetics, resulting in the alternative regulation of cancer associated gene expression.

Inflammation leads to oxidative damage in the DNA of the infected cell, due to the response of immune cells. NF-κB and STAT3, which are activated by proinflammatory cytokines and chemokines during chronic inflammation, can cause the parenchymal cells to produce excess amount of reactive oxygen species (ROS) and reactive nitrogen species (RNS), which induce genomic instability and DNA mutations [63,64,65]. Mutations and chromosomal changes are thought to be involved in tumor progression, which may be accelerated by a chronic inflammatory microenvironment, which accumulates mutations as a result.

## 3. Stem Cell Niche and Cancer-Inducing Niche

Although the initial concept of stem cells is more than 100 years old, and its biology was largely discovered during the previous semicentennial period, much about their nature remains unknown. Stem cells are critical for tissue homeostasis; they are responsible for tissue regeneration, which replaces dead cells with new cells according to cellular senescence. To maintain tissue homeostasis through their lifetime, stem cells must keep an accurate balance between self-renewal and differentiation. The essential mechanisms must be maintained in a delicate balance, together with the direction of differentiation. These mechanisms must be understood in order to determine how stem cells are regulated and contribute to human health and diseases [66,67]. Recent studies have revealed that the microenvironment, or so called “niche,” is significant in various ways for stem cells.

The concept of niches specific to stem cells was first introduced almost five decades ago by Schofield, who theorized that stem cell properties are dependent on their microenvironment [68]. Niche has usually been defined as the location surrounding the stem cells. However, this definition of niche is being changed to involve the cellular components, such as fibroblasts, endothelial cells and immune cells, which are rich in extracellular matrices, and secreting factors and receptors as well as the signs of the microenvironment affected by the metabolism [69,70]. Secreted factors bound to stem cell surface receptors stimulate the intracellular signaling cascade in order to direct stem cell fates by controlling self-renewal and differentiation [71,72,73]. Therefore, the concept of seed and soil is still feasible, with, the microenvironment significantly involved in the cancer development [74]. Chronic inflammatory conditions, as the surrounding niche, trigger stem cells to develop cancer [75,76]. In this process, the CSCs may appear from the normal stem cells affected by the cancer-inducing niche. Characterization of the cancer-inducing niche will be the most important key to establishing the logic of CSC development from normal stem cells.

**Figure 4:**
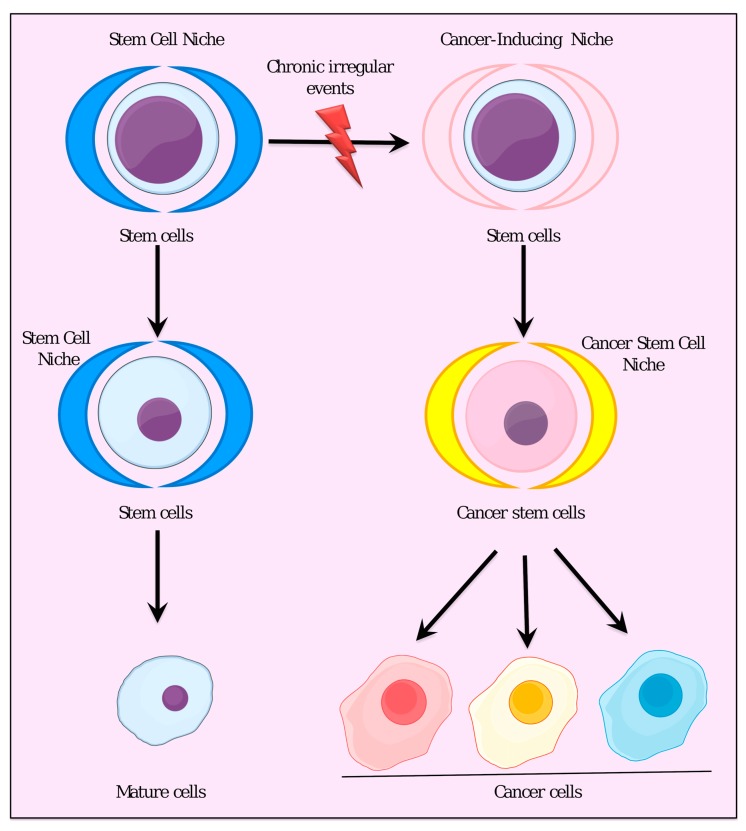
Stem cell differentiation in the stem cell niche and cancer-inducing niche.

The cancer stem cells concept is greatly changing the historic viewpoint on cancer, namely mutation theory, which is now being challenged due to its failure to account for determinants of cancer induction [77]. The cancer-inducing niche can be explained as the continuous irregular environment that we typically recognize as chronic inflammation (Figure 4). This chronic inflammation can be explained by the primary role of cytokines and other soluble mediators in the tumor microenvironment. However, many questions remain to be answered. What are the exact components of the cancer-inducing niche? Do they vary according to the type of cancer? What kinds of differences lie between the cancer-inducing niche and the normal stem cell niche? In the next couple of years, these critical questions will be answered by scientific researchers employing the new strategies that will become available as stem cell technologies advance.

After CSC development, CSCs will establish their own niche as the CSC niche. Cells composing the CSC niche are not only necessary for the maintenance of CSCs, but also for the generation of factors and tumor associated cells that maintain the properties of CSCs, including invasion, metastasis, and promotion of angiogenesis [78,79,80]. The CSC niche contains cellular components such as cancer associated fibroblasts [81], tumor associated macrophages [82,83]. tumor associated neutrophils [84], MSCs [85], and cell-mediated adhesion [86] and soluble-factors, which play critical roles in cell–cell communication. The niche before the CSC niche, as a cancer-inducing niche, should be distinguished from the CSC niche. The cancer-inducing niche can be studied experimentally only by the conversion of ESCs or iPSCs to CSCs.

## 4. Inflammatory Microenvironment Stimulates the Generation of Cancer Stem Cells

**Figure 5:**
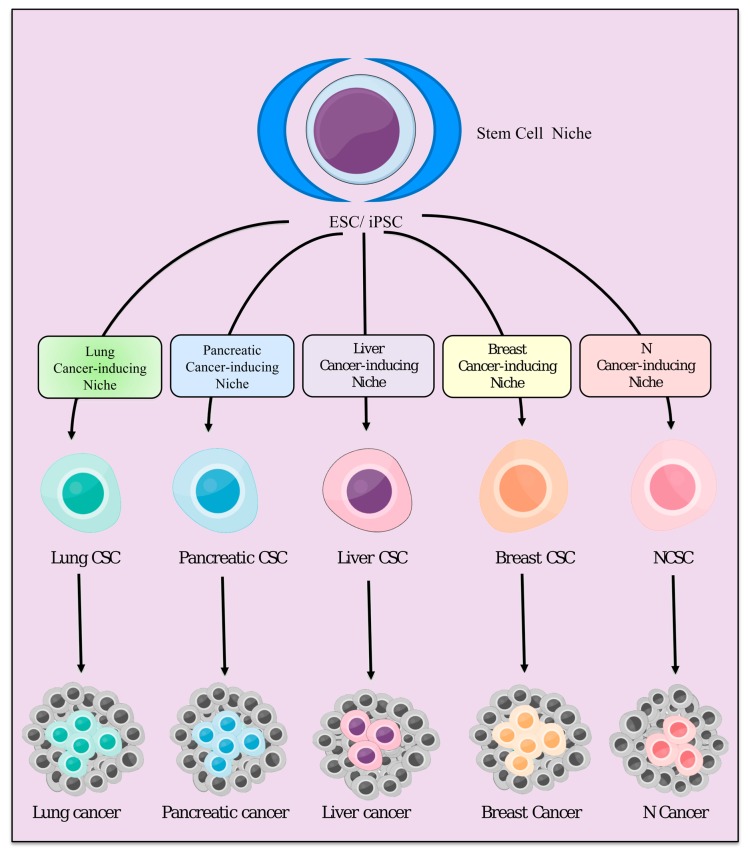
The hypothesis of stem cell (ESCs/iPSCs) differentiation under different cancer-inducing niches.

We hypothesize that chronic inflammation stimulates the generation of CSCs. According to our concept of the cancer-inducing niche, being developed from normal stem cells in chronic conditions, CSCs are likely to be progenitor cells, which are destined to become cancer cells (Figure 5). Once CSCs have developed, the CSC niche, together with the cancer-inducing niche, provides a suitable microenvironment to maintain CSCs, which in turn develops malignant tumor. The phenotype of the malignant tumor appears to depend on both the tissue specific microenvironment and the CSC niche, as demonstrated by previous experiments [87,88]. We have demonstrated that iPSCs could acquire characters of CSCs when iPSCs were cultured in the presence of a conditioned medium prepared from various cancer cell lines [87,89].

### 4.1. Lung Cancer Stem Cells

Lung cancer is the most common cause of cancer-related death worldwide [90]. It is a heterogeneous disease with two different pathological classes: non-small cell lung cancer (NSCLC), which makes up 80% of cases, and small cell lung cancer (SCLC), which makes up 20% of all lung cancers [91]. The first experimental evidence for the presence of a stem-like subpopulation in lung cancer was demonstrated by Carney [92,93]. Carney and colleagues described a rare population (<1.5%) of cells in adeno-carcinoma and SCLC specimens, with the ability to form colonies in soft agar and to form malignant tumors, the characteristics of which were identical to those of the original SCLC and adenocarcinoma tumors, upon injection of the cells into nude mice. After that, scientists have tried to define lung CSCs using various experimental methods. During the course of time, lung CSCs have been isolated from lung cancer derived cell lines H460, H23, HTB-58, A549, H441, and H2170 cells, which have high tumorigenic potential in vivo [94]. Simultaneously, lung CSCs isolated from patient primary tumors exhibited high resistance to standard anticancer agents in vitro [95]. The presence of lung CSCs helps in understanding the phenotype and characters of the sub-population, but does not explain the origin. On the basis of our approach, iPSCs can be converted into lung CSCs, which are very important to describe the origin of lung CSCs, as well as the process of the mechanism of conversion.

Based on the cancer-inducing niche hypothesis, our group established a CSC model from mouse iPSCs through epigenetic regulations affected by the conditioned medium (CM) from Lewis lung carcinoma (LLC) cells for four weeks without any genetic manipulation [89,96,97]. These cells exhibited self-renewal potential by their capacity of spheroid and differentiation potential of some specific phenotypes. Furthermore, these cells sustained expression of genes associated with the stem cell character and undifferentiated state, such as Nanog, Sox2, Oct4, and c-Myc. Simultaneously, the tumorigenic potential was confirmed by the development of malignant tumors, which were recognized by glandular epithelial hyperplasia, a high nuclear to cytoplasmic ratio, severe nuclear atypia, multiple pathological mitotic figures, micro metastases, and hypervascularization, indicating angiogenesis [89].

### 4.2. Pancreatic Cancer Stem Cells

Pancreatic ductal adenocarcinoma (PDAC), the most common type of pancreatic cancer [98], which constitutes 90% of pancreatic cancers, is one of the most aggressive solid malignancies [99]. PDAC solid tumors are comprised of a wide range of heterogeneous populations of cells, including CSCs [100]. Rapid progress in identification and isolation of pancreatic CSCs has facilitated the generation of new mouse models [101,102]. Unfortunately, most of these models are genetically engineered, and may not be suitable to understand spontaneous tumor occurrence. In contrast, a novel trial to produce pancreatic CSCs from iPSCs has been explored by the exposure to CM of two different pancreatic carcinoma cell lines: PK8 and KLM1 cells [87]. The microenvironments provided by the pancreatic cancer cell lines successfully converted iPSCs into CSCs. The two new pancreatic CSCs were subcutaneously transplanted into immunodeficient Balb/c nude mice. Approximately 1 month later, miPS-PK8cm cells and miPS-KLM1cm cells generated tumors in 9 out of 9 mice for each cell line, indicating tumorigenic potential. The histology of the tumors showed the characteristics specific to the PDAC phenotype. Among the epithelial-like structures, pancreatic intraepithelial neoplastic lesions (PanIN) were found, together with a moderate figure of poorly differentiated ductal structures, in the tumors derived from both miPS-PK8cm and miPS-KLM1cm cells. These cells were found to be metastatic to the liver, while the metastatic nodules exhibited the phenotype of teratocarcinoma, containing very few structures corresponding to PDAC. Under treatment with puromycin, the pancreatic CSCs in the primary cultures of the PDAC-like tumor were able to be selected without the intrusion of cells from the host. The pancreatic CSCs, which were enriched from the first primary culture, generated a specific cell population of well-defined colonies surrounded by myofibroblast-like cells, most likely pancreatic stellate cells. The pancreatic CSCs from the primary culture exhibited the specific feature of generating a spontaneous self-supporting system for integrity and maintenance, implying their enhanced malignancy, which was not observed in iPSCs. This primary culture showed significant elevation of CSC markers, such as CD24a, EPCAM, and CD133, compared to iPSCs.

### 4.3. Liver Cancer Stem Cells

Liver cancer, also known as primary hepatic cancer, is a cancer that occurs in the liver. Liver cancer is the second most frequent cause of cancer-related death [103]. In liver cancer, accumulating evidence has confirmed the presence of a small subcategory of cancer cells with stem cell properties, which are recognized as liver CSCs [104,105]. Liver cancer was formerly considered to be a disease derived from dedifferentiation of hepatocytes. However, due to the enormous progress in stem cell research during the last couple of years, liver cancer is currently believed to be derived from transformed liver stem cells [106,107]. Therefore, any proliferative cells in the liver may be subjected to malignant transformation, which is considered one possible origin of liver CSCs. However, the question is, “How does this transformation happen?”

Many types of liver cancer will develop as a result of chronic hepatitis that is initiated by viral infection, too much alcohol, or non-alcoholic fatty liver disease. During this process, the microenvironment will initiate and promote liver cancer [108]. Furthermore, this process may be explained by the transformation of liver stem cells into liver CSCs [109,110]. To explain this process of liver CSC development more precisely, our group is currently working on the conversion of iPSCs into liver CSCs with the CM from human hepatocellular carcinoma cell line Huh7 cells [88]. The malignant tumor derived from the converted cells demonstrated high expression of glypican-3, alpha fetoprotein, and cytokeratin19, which were considered liver cancer associated markers [111,112], as well as CD44, CD133, and CD24, which were considered LCSC markers [113,114,115]. This model will be very important and useful to identify the significant molecular mechanisms required for liver CSC development, as well as to develop effective therapy for liver cancer.

### 4.4. Prostate Cancer Stem Cells

Similar to most solid tumors, prostate cancer is heterogeneous, containing many phenotypes of carcinoma cells. The first experimental proof for the presence of prostate CSCs was reported by three independent groups in 2005 [116,117,118], who manipulated different ways to isolate self-renewing and tumorigenic cells from prostate tumors in patients and prostate cancer cell lines. Some scientists hypothesized that prostate CSCs could be developed from cell fusion between stem cells and surrounding cells, induced by irregular conditions [119]. This cell fusion may result in the combination of abnormally differentiated cells with self-renewal properties of normal stem cells to accomplish neoplastic transformation. Recently, prostate CSCs were reported to be derived from the malignant transformation of normal stem cells [120]. Very recently, prostate cancer stem-like cells were developed from mouse iPSCs, using CM derived from mouse prostate cancer cell line RM9 cells [121]. In this study, miPSCs were induced to convert into prostate CSCs after treatment for 6 weeks. The converted cells, named miPS-RM9cm cells, were subcutaneously injected into male C57BL/6 mice (6 to 8 weeks old) and monitored for 2 weeks. As a result, malignant tumors were generated in 5 out of 5 mice after injection of the cells. miPS-RM9cm cells expressed a high level of the CSC marker CD44, indicating that miPSCs had obtained malignancy after the treatment with CM.

From human iPSCs, we have developed CSCs with the conditioned media of several cancer cell lines [122]. These cells commonly expressed human embryonic stem cell (hESC)/hiPSC-specific genes (POU5F1, SOX2, NANOG, LIN28, and SALL4) at a level equivalent to those of the control hiPSC 201B7. The CSCs could be divided into three groups based on their culture conditions and original cancer tissues from their gene expression profiles.

Collectively, it is conceivable to suppose the secretion of pro-inflammatory and/or inflammatory cytokines, such as IL-6, IL-8, IL-17, TNF-alpha, IFNs, and so on, from the cancer cell lines and conditioned medium, could be enriched with the cytokines, which could be used for the conversion of iPSCs into CSCs. In this context, the cancer-inducing niche could experimentally be provided by the conditioned medium from cancer derived cell lines.

## 5. Epigenesis or Mutagenesis?

### 5.1. Hyper- and Hypo- Methylation of DNA

Epigenetic events, such as DNA methylation, chromatin remodeling, noncoding RNAs, and histone modifications, control gene expression [123], which determines the cell fate without changing DNA sequences [124]. These events are pivotal during normal mammalian development [125]. Disruption of normal epigenetic processes leads to significant change of the cell fate from normal to malignant. These epigenetic changes are believed to be responsible for the source of diversity in cancer [126]. The chronic stimulation of the cancer-inducing niche affects the normal SC altering the molecular signaling pathways; this abnormality could be explained by perturbed epigenetics. The accumulation of such epigenetic abnormalities has been suggested to be an early event that prompts and gives rise to CSCs [127]. Now, there is a strong argument about the source of CSCs, whether they originate from adult SCs, from progenitors, or from differentiated cells that have dedifferentiated to acquire self-renewal ability. Regardless of these different routes, the altered epigenetics represent the first event of the generation of CSCs [128,129]. DNA methylation is a main epigenetic modification that powerfully controls gene expression. The patterns of DNA methylation in cancer cells are well known to be distinguished from normal cells [130]. Most tumors display a global hypomethylation that causes chromosomal instability and re-expression of silenced genes that play critical roles in tumorigenesis [131,132]. Furthermore, most tumors exhibit DNA hypermethylation that leads to suppression of tumor suppressor genes [133]. The suppression of these tumor suppressor genes may lead to the formation of CSCs [134,135].

Regarding the proposed model of CSCs generated from iPSCs with the CM of lung cancer derived cells, regulation of gene expression by epigenesis rather than by mutagenesis may perform a critical role in the CSCs’ initiation [89,97]. In this model, DNA hypomethylation was considered to be the main cause in the conversion of iPSCs into CSCs. The differentially methylated regions were identified, and hypomethylation was found to be greater than hypermethylation in the CSCs when compared to miPSCs. Further bioinformatics analysis of KEGG pathways identified hypomethylated genes in the category of the ‘PI3K-Akt pathway.’ The expression of the nominated genes in the category was then assessed to find the responsible gene. Finally, PI3 kinase genes were found to be overexpressed, resulting in the constitutive activation of the PI3K-Akt-mTOR signaling pathway, which was considered to be a critical effector of carcinogenesis in several cancer types [136,137]. From these results, we successfully demonstrated that the epigenetic changes were likely the first step in CSC development from iPSCs, and that they have a critical role in CSCs as well as tumor initiation.

### 5.2. DNA Mutations

There is no doubt about the presence of mutations in cancer cells, and much evidence has been reported that the mutation or gene introduction induces the transformation of the cells. However, no evidence is available to demonstrate cancer initiation or CSC generation by mutation. Since cancer initiation is more important than cancer progress, we should identify the critical events happening in cancer initiation. To date, scientists have explained that mutations were mainly responsible for cancer progression [138], while initiation could not be explained by mutation [139]. Furthermore, they demonstrated that a mutation alone is unlikely to cause cancer, has a limited tendency to cause cancer, and is unable, by itself, to transform a normal cell to a malignant cell. They hypothesized that signs of transformation were perceived as DNA mutations after the cells changed from a normal state to a cancerous state. They tried to explain that the phenotypes of cancer cells could be determined by the order and points of mutations. The mutations, which have long been studied as the effectors and the initiators of cancer, were considered to be the result of cellular transformation from normal to malignant [140]. Consequently, the observed mutations in cancer cells were understood to produce the malignancy.

Regarding cancer stem cell initiation from iPSCs, our group demonstrated that pancreatic cancer stem-like cells, derived from iPSCs in the presence of CM of pancreatic cancer cell lines, represent a realistic model for pancreatic CSCs, which provides a PDAC phenotype [87]. Furthermore, it was mentioned that the conversion process of iPSCs has not been achieved under any genetic manipulation, and, after analysis, the expected mutation in this model was not found. This result suggests that the epigenetic events from chronic stimulation are likely to be the critical event in the conversion of iPSCs to CSCs, and that mutation is not the cause of cancer initiation, but a result associated with the cancer progress at the later stages of cancer development.

## 6. Conclusions

Scientists in cancer research continuously provide new evidence of the critical events that initiate cancer. Chronic inflammation has been considered to be the initial step of cancer stem cell generation, as well as cancer initiation. However, the critical point of initiation has long been controversial. Due to the recent availability of ESCs or iPSCs, the initiation of CSCs has made it possible to demonstrate the presence of a cancer-inducing niche as a quintessential factor of cancer initiation without mutations. Researchers will soon identify the inflammatory factors under chronic situations and signal transduction paths closely related with the initiation of CSCs. This will help us study how to defeat cancer in the future.

## Abbreviations

CSCs	Cancer stem cells
iPSCs	Induced pluripotent stem cells
CICs	Cancer-initiating cells
ROS	Reactive oxygen species
DCs	Dendritic cells
ESC	Embryonic stem cell
MSCs	Mesenchymal stem cells
CM	Conditioned medium
PDAC	Pancreatic duct adenocarcinoma
ROS	Reactive oxygen species
SCLC	Small cell lung cancer
